# *Withania Somnifera* (Ashwagandha) and Withaferin A: Potential in Integrative Oncology

**DOI:** 10.3390/ijms20215310

**Published:** 2019-10-25

**Authors:** Rinku Dutta, Roukiah Khalil, Ryan Green, Shyam S Mohapatra, Subhra Mohapatra

**Affiliations:** 1 Department of Molecular Medicine, Morsani College of Medicine, University of South Florida, Tampa, FL 33612, USA; rinku@usf.edu (R.D.); roukiah@usf.edu (R.K.); rjgreen@usf.edu (R.G.); 2 Center for Research and Education in Nanobioengineering, Morsani College of Medicine, University of South Florida, Tampa, FL 33612, USA; smohapat@usf.edu; 3 Department of Internal Medicine, Morsani College of Medicine, University of South Florida, Tampa, FL 33612, USA; 4 James A Haley VA Hospital, Tampa, FL 33612, USA

**Keywords:** cancer, mechanism of action, *Withania Somnifera*, Withaferin A

## Abstract

Ashwagandha (*Withania Somnifera, WS)*, belonging to the family Solanaceae, is an Ayurvedic herb known worldwide for its numerous beneficial health activities since ancient times. This medicinal plant provides benefits against many human illnesses such as epilepsy, depression, arthritis, diabetes, and palliative effects such as analgesic, rejuvenating, regenerating, and growth-promoting effects. Several clinical trials of the different parts of the herb have demonstrated safety in patients suffering from these diseases. In the last two decades, an active component of Withaferin A (WFA) has shown tremendous cytotoxic activity suggesting its potential as an anti-carcinogenic agent in treatment of several cancers. In spite of enormous progress, a thorough elaboration of the proposed mechanism and mode of action is absent. Herein, we provide a comprehensive review of the properties of *WS* extracts (*WS*E) containing complex mixtures of diverse components including WFA, which have shown inhibitory properties against many cancers, (breast, colon, prostate, colon, ovarian, lung, brain), along with their mechanism of actions and pathways involved.

## 1. Introduction

Ashwagandha (*Withania Somnifera, WS)*, belonging to family Solanaceae, is an Ayurvedic herb also known as Indian winter cherry and Indian ginseng that has been traditionally known since ancient times in India for its numerous beneficial health activities. *WS* is one of the most important herbs in Ayurveda, which has been used for >3000 years in stress management, energy elevation and improving cognitive health [1,2,3,4] and to lower inflammation, blood sugar levels, cortisol, anxiety, and depression [5,6]. The plant is an erect, grayish, evergreen shrub with long tuberous roots, short stems, ovate and petiolate leaves, and greenish axillary and bisexual flowers. The leaves, roots, stems and flowers bear medicinal values with 29 common metabolites derived from the leaves and root extracts [6,7]. To date, this medicinal plant has been found to have anti-epileptic, anti-inflammatory, anti-arthritic, anti-depressant, anti-coagulant, anti-oxidant, anti-diabetic, anti-pyretic efficacies along with palliative effects such as analgesic, rejuvenating, regenerating and growth-promoting effects [8].

Despite its medical use from time immemorial in many parts of the world, the basic and mechanistic studies relating to the potential of *WS* extracts (*WS*E) has not been studied in the clinical realm until relatively recently. Thus, several additional Randomized Double-blind Placebo Control Trials have been formed for various clinical conditions (Table 1) ranging from weight management to schizophrenia. So far each of these studies showed significant effectiveness of *WS* used for intervention vs. control group. Most notably, together these studies revealed that in all of these studies *WS* was safe and tolerable (Table 1).

In contrast to these illnesses, the role of *WS* in cancers was reported around 1992 [15]. *WS* was shown to impede the growth of new cancer cells, but not normal cells, help induce programmed death of cells by generating reactive oxygen species (ROS), and sensistize cancer cells to apoptosis [16,17,18]. Pre-clinical studies in several cancer types have shown up to 80% inhibition using combination chemotherapy [19]. Despite this progress, however, a comprehensive review of molecular mechanisms of the regulation by *WS* and its major component Withaferin A (WFA) is lacking. Herein, we provide a comprehensive review of the effects of WS vs. WFA on different cancers as well as their mechanistic role in decreasing the cancer growth and reducing toxicities resulting from radio and chemotherapies.

## 2. History of *WS*

Herbal therapies have been extensively used in traditional medicine (including Ayurvedic and Chinese) since time immemorial. Medicinal plants contain different cytotoxic constituents that induce autophagy, necroptosis and apoptosis by influencing various proteins involved in the apoptotic pathway [20]. Although the various beneficial effects of the *WS* plant and its root, stem and leave extracts are known historically, the first published literature on the antibacterial principle of *WS* dates back to 1958 by Kurup PA [21]. Later, Malhotra et al. reported the effects of the total plant extract on central nervous system, smooth muscles, cardiovascular system, respiration and skeletal muscles in the 1960s [22,23].

The chemotherapeutic properties of the substances isolated from the leaves was found in the literature but it was Dhalla et al. who first reported the chemical studies of the leaves of *WS* [24,25]. In 1973, the root extract of *WS* was isolated as a C28 steroid lactone as 5, 20α-Dihydroxy-6α, 7α-epoxy-1-oxo-with a-2, 24-dienolid whose structure was found to be similar to a withanolide isolated from the roots of Withania coagulants [26].

For the first time, Chakraborti et al. reported the variations in the antitumor constituents of the *WS* dunal and the in vivo growth inhibitory effects of the root extracts of plant in a transplantable mouse tumor, Sarcoma 180 in 1992 [15,27]. Thus, intraperitoneal injection of the root alcoholic extract 400 to 1000 mg/kg body weight in BALB/c mice daily post 24 h intradermal inoculation of 0.5 × 10^6^ cells of S-180 resulted in tumor regression. Later the same group showed the radio-sensitizing and antitumor effects of the root extract in the sarcoma model [28]. It was not until 1996, that WFA’s radiosensitizer activity was reported that caused V79 cell survival reduction where 1-h pre-treatment at 2.1 µM dose before radiation significantly killed cells [18,29]. Later, the anti-carcinogenic activities of WFA was found to be effective in various types of cancer treatment both in vitro and in vivo.

## 3. *WS* Extracts and their Anticancer Activity

The pharmacological activity of the commercially available herbal supplements of *WS* extracts is conferred by its various alkaloids as well as WFA. Since the European Food Safety Authority (EFSA) has classified WFA as toxic, its application in cancer therapy by killing tumor cells is immense. Based on the types of extracts of the different parts of the plant such as water extract, methanol/ethanol extracts mainly from leaves, stems and roots research has advanced in exploring the active constituents and their effects in cancer. Table 2 summarizes these plant parts and their efficacies in cancer therapy. Interestingly, the whole plant extract was found to increase cell proliferation, stem cell proliferation, WBC (white blood cells) content, in sharp contrast to using either root or stem extracts. The method of extraction i.e., using organic solvents such as methanol or aqueous extracts for either roots, stems or leaves did not change the anti-cancer mechanism of the extracts. Radio-sensitization to altered expression of inflammatory cytokine genes, enhancing generation of reactive oxygen species, inhibiting NF-κB activation. Notably, leaf extracts showed alteration of genes involved in cell cycle.

## 4. Active Components in *WS* Extract

**Figure 1:**
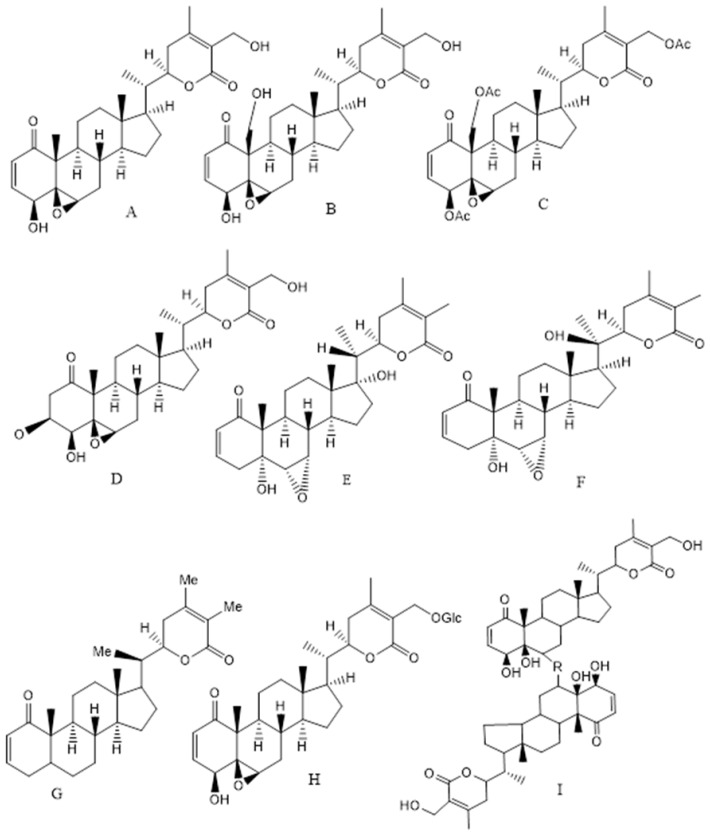
Structure of the Ashwagandha extract compounds from leaves, roots, stem and fruits (**A**) Withaferin A, (**B**) Withalongolide A, (**C**) Withaferin triacetate, (**D**) 2,3-Dihydro-3β-methoxy Withaferin A, (**E**) Withanone, (**F**) Withanolide A, (**G**) Withanolide D, (**H**) Sitoindoside (IX) or 27-O-glucopyranosyl withaferin A and (**I**) Thiowithanolide (R = S and S = O).

The major biological compounds, which are found from different parts of the plant, are C-28 steroidal lactone triterpenoids, also known as withanolides (approx. 40 unique compounds), which are mostly comprised of withanolide A, WFA, withanone and withanolide D. The structure of withanolides is based on an ergostane backbone comprised of a lactone ring at the C-8 or C-9 side chain. Apart from withanolides, alkaloids, flavonoids, steroids, withanamides, withanosides, withanolide glycosides with a glucose at carbon 27 also known as glycol-withanolides (sitoindoside IX and X), steroidal saponins containing an additional acyl group (sitoindoside VII and VIII), cuscohygrine, anahygrine, salts, coagulins and other nitrogen containing compounds are also found in the various plant parts. Alkaloids, for example isopelletierine, cuscohygrine, anahygrine, tropine, and withanine, are relevant phytochemicals of *WS*. Apart from the broad-spectrum therapeutic activity, the extracts of the leaves, roots, stems and fruits as well as the isolated withanolides, Withaferin, have emerged as a potent anti-carcinogenic agent in lung, breast, colon, cervical, brain, prostate and other cancers. Particularly, WFA, Withanolide D, Withalongolide A and its triacetate derivatives have been found to possess anti-carcinogenic activities (Figure 1) [54,55]. WFA acts as an inhibitor of the chaperon p97 and it along with its analogues can be a proteostasis modulator by retaining p97 activity and cytostatic activity in vitro [54]. Recently, Motiwala and co-authors have reported the synthesis and cytotoxicity of semisynthetic Withalongolide analogues where 24 compounds were tested on five cell lines (JMAR, MDA-MB-231, SKMEL-28, DRO81-1, and MRC-5) [55]. The other constituents including WFA have hepatoprotective, cardio-protective, immunosuppressive, anti-inflammatory, neuroprotective, anti-oxidative and anti-microbial activities. WFA treatment leads to apoptosis, evasion of anti-growth signaling and immune system along with sustained proliferative signaling and interactions with the tumor microenvironment [56]. The recent updates on the anti-carcinogenic effects of WFA on various cancers (breast, colon, prostate, lung, ovarian along with renal, head and neck, pancreatitis, liver and skin cancers) are summarized in Table 3 along with their mechanisms of action and plausible pathways.

## 5. Role of *WS* and WFA in Various Cancers

### 5.1. Breast Cancer

Luminal A/B (estrogen-receptor and/or progesterone-receptor positive and *HER2* (human epidermal growth factor receptor 2) negative or positive) and triple negative/basal like (TNBC) (estrogen-receptor, progesterone-receptor and HER2 negative) are the molecular subtypes of breast cancers where the effects of WFA have been extensively studied [64,65]. When studied for the proapoptotic response of WFA, it was found the phytochemical downregulated the estrogen receptor-α (ER-α) protein in MCF-7 cells. This effect reversed in presence of 17β–estradiol (E2). Thus WFA acts as anti-estrogen and p53 knockdown partially reduce WFA-mediated proapoptotic effects [66]. Moreover, in therapy-resistant TNBC, WFA studied for invasive and metastatic effects showed not only anti-metastatic behavior in nM concentrations but also lower extracellular matrix (ECM) gene expression and transcriptional patterns towards non-invasiveness by targeting uPA (urokinase-type plasminogen activator) signaling cascade [67]. In cancer patients using cancer genome atlas, WFA was found to suppress TNBC gene expression compared to the luminal cancers [68]. These studies provide invaluable evidence of the anti-cancer effects of WFA in both luminal and TNBC via diverse pathways.

Although *WS* leaf, root and fruit extracts have shown curative effects in multiple diseases, the exact mechanism behind the action of these extracts is not well understood. Mohan et al. [69] were the first to report that WFA can bind to vimentin intermediate filaments causing them to aggregate in the cytoplasm leading to apoptosis in the MCF-7 cell line. Yang et al. later reported that treatment with the root extract leads to inhibition of mammary cancer metastasis and epithelial to mesenchymal transition via vimentin inhibition [70].

Widodo et al. reported that WFA selectively activated p53 in tumor cells treated with the leaf extract of Ashwagandha [71] leading to growth arrest and apoptosis. Amongst other mechanisms, apoptosis due to generation of reactive oxygen species (ROS) by WFA has been widely reported. Hahm et al. demonstrated both *in vitro* and *in vivo* that the role of WFA in inducing apoptosis is mediated by ROS generation due to the inhibition of mitochondrial respiration. MDA-MB-231 and MCF-7 cell lines showed increased ROS production upon treatment as opposed to the normal human mammary epithelial cells (HMEC) [72] which did not increase ROS production. The molecular phenomenon behind the WFA-induced ROS-mediated apoptosis was due to the inhibition of oxidative phosphorylation and complex III activity accompanied with apoptotic histone-associated DNA fragment release in the cytosol as evidenced by significant reduction in the ectopic expression of Cu, Zn-superoxide dismutase in the aforementioned breast cancer cell lines. This mechanism was tested in mitochondrial DNA-deficient Rho-0 variants of MDA-MB-231 and MCF-7 cells where no apoptosis or related mitochondrial stress were observed [72]. In another study, Ghosh et al. demonstrated that not only apoptosis, but also paraptosis (non-apoptotic programmed cell death) is caused by WFA-induced production of ROS. These observations were supported by the formation of large cytoplasmic vacuolar structures due to the fusion of mitochondria and endoplasmic reticulum dilation in human breast cancer cell lines (MDA-MB-231 and MCF-7) along with downregulation of the endogenous paraptosis inhibitor, Actin Interacting Protein-1 (Alix/AIP-1), upon WFA treatment [73].

**Figure 2:**
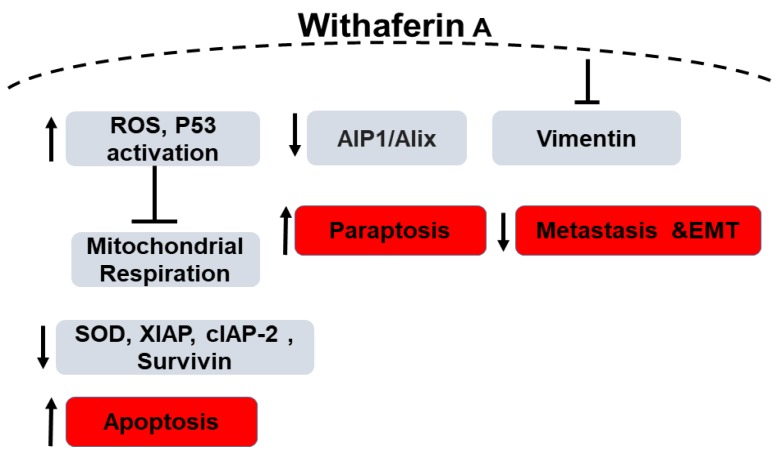
Schematic diagram of the role of Withaferin A in Breast Cancer. “↑” denotes an increase, “↓” denotes decrease and “T” denotes inhibition.

Widodo et al. have used hammerhead ribozymes (catalytic RNAs) to identify genes and targets involved in WFA-mediated cellular cytotoxicity. MCF-7 breast cancer cells were infected with a retroviral vector carrying a randomized ribozyme library. The cells were then treated with WFA and ribozymes were retrieved from the surviving cells. Targets identified were validated using shRNA knock down of the target genes as well as bioinformatics pathway investigation. shRNA studies have shown 4 genes (*TPX2* (Targeting protein for Xklp2), *ING1* (inhibitor of growth protein 1), *TFAP2A* (transcription factor AP-2 alpha) and *LHX3* (LIM/homeobox protein) are involved in WFA and withanone induced cellular cytotoxicity. Silencing these four genes led to decreased killing of cancer cells by 20–40% by the extract. Using bioinformatics and systems biology approach, the group identified p53 and apoptosis pathways to be involved in WFA-mediated cytotoxicity (Figure 2). Network interaction analysis showed 4 gene clusters: *CDK4* (cyclin-dependent kinase 4), *TFAP2A*, *CDKN1A-p21* (cyclin dependent kinase inhibitor 1A) and *ING1* linked by *p53* and *PCNA* (proliferating cell nuclear antigen). They hypothesized that the extract-mediated cellular cytotoxicity through mitochondrial stress and DNA damage pathway leads to activation of ROS-mediated cellular signaling. The group found an increase in γ-H2AX and number of cells expressing the phosphorylated form which is a marker for DNA damage in WFA treated MCF-7 cells. In addition, an increased tolerance to WFA treatment on p21^-/-^ cells confirmed the role of CDKN1A-p21 in WFA-mediated cytotoxicity. ROS was detected in MCF-7 cells treated with the extract, withanone or withaferin. As ROS is well known to affect mithochondrial membrane potential, they found a change in mitochondrial membrane potential and altered mitochondrial morphology in WFA treated cells. Therefore, the study concludes that Ashwagandha extract and Withanone mediate selective killing of cancer cells by induction of ROS production and mitochondrial damage and hence, can be used for effective and safe cancer therapy [74]. Recently, it has been reported that mitochondrial dynamics are involved in breast cancer apoptosis when treated with WFA. [75]. Additionally, although the levels of XIAP (X-linked inhibitor of apoptosis protein), cIAP-2 (cellular inhibitor of apoptosis protein-2) and Survivin proteins were found to be reduced in MDA-MB-231 and MCF-7 cells when treated with WFA, in a MDA-MB-231 xenograft model, WFA-mediated inhibition was associated only with Survivin protein suppression thus highlighting the importance of Survivin suppression in WFA-induced apoptosis. These results provided a novel insight into the molecular mechanism of WFA-induced apoptosis in human breast cancer cells [76].

### 5.2. Colorectal Cancer

Colorectal cancer (CRC) is divided into inherited, familial and sporadic types, which represents 70% of all CRC cases. Histologically, CRC is classified into adenocarcinoma (representing 95% of all cases), lymphoma and squamous cell carcinoma. CRC usually develops from pre-neoplastic lesion due to 2 major genetic alterations either chromosomal instability or microsatellite instability. The major molecular mutations commonly found in CRC include, p53 mutations (50%), *KRAS* (K-ras) mutations (25–60%), *BRAF* (B-Raf) (10%) and *PIK3CA* (phosphatidylinositol 3-kinase catalytic subunit alpha) (10–30%) [77].

Whereas the role of WFA has been examined in colorectal cancer cells, the investigations using *WS* are limited despite the latter’s use as a dietary supplement, i.e., it is taken orally. Major component of *WS* was found to have an anti-carcinogenic effect on colorectal tumors through alterations of multiple signaling pathways. The anti-cancer effects of WFA on the proliferation and migration of colorectal cancer cell lines have been cited due to reduced transcriptional activity of STAT3 (signal transducer and activator of transcription 3). Also, in HCT116 xenograft tumors in a Balb/c nude mouse model, the authors found a regression in the growth of the tumors thus proving the potential of WFA as a STAT3 inhibitor [78]. Further, Notch-1 signaling pathway plays a crucial role in the development of colon cancer and WFA has been shown to inhibit this signaling including Akt/NF-κB/Bcl-2 pro-survival pathways. A molecular link between Notch/Akt/mTOR signaling was established and WFA inhibition of Notch-mediated signaling aided in JNK-( c-Jun N-terminal kinase) mediated apoptosis in colon cancer cell lines, HCT-116, SW-480 and SW-620 [79,80].

The chemopreventive effects of WFA have been studied by Chandrasekaran and colleagues in spontaneous and inflammation-associated colon cancer transgenic adenomatous polyposis coli (*APC^Min/+^*) and azoxymethane/dextran sodium sulfate (AOM/DSS) induced mice models respectively. WFA was orally administered at doses of 3 and 4 mg/kg and the authors found 59% reduction of tumor and polyp initiation and progression in the WFA treated mice compared to the controls [80]. WFA downregulated expression of inflammatory markers in these tumors such as IL-6, TNF-α, COX-2 along with pro-survival markers such as pAkt, Notch1 and NF-κβ [80]. These results are in agreement with the priming effect of the root extract of the herb in chemotherapy that modulated mitochondrial function, thus proving the priming effect of the root extract as a potential mechanism through increased ROS [33].

### 5.3. Prostate Cancer

**Figure 3:**
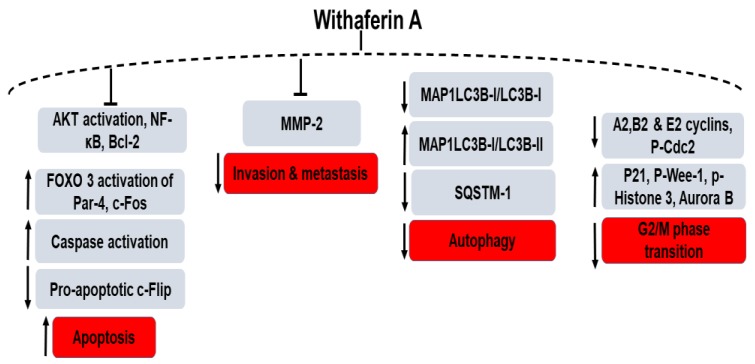
WFA’s role in the various signaling pathways of prostate cancer. “↑” denotes an increase, “↓” denotes decrease and “T” denotes inhibition.

WFA has been utilized as a therapeutic agent for prostate cancer therapy where it acts as a regulator of G2/M phase transition of the cell cycle through the upregulation of phosphorylated Wee-1, phosphorylated histone H3, p21, Aurora B and the downregulation of A2, B1 and E2 cyclins and phosphorylated Cdc2 (Tyr15) [81]. Among various mechanisms involved in prostate cancer initiation and progression, activated protein kinase B/Akt plays a key role where the inactivation of the tumor suppressor *PTEN* gene (phosphatase and tensin homologue) leads to activation of Akt and subsequent development of prostate tumors [82] (Figure 3). Moselhy et al. reported the chemopreventive action of WFA in the *Pten* conditional knockout mouse (*Pten*-KO) model with constitutively activated Akt signaling. Oral administration of WFA with a dose of 3–5 mg/kg inhibited the activation of Akt and facilitated the FOXO3a-(Forkhead box O3a) mediated activation of Par-4 (prostate apoptosis response-4) leading to delayed tumor progression in preclinical prostate cancer models. Thus, WFA has been effective in up-regulating Par-4 and FOXO3a proteins (PI3K/Akt pathway regulated) in *Pten*-KO mice with a promising outcome for patients with Akt-activating mutations [83,84].

In prostate cancer cells, switching from autophagy to apoptosis has been found after treatment with a semi-synthetic analogue, 3-azido derivative of WFA (3-AWFA) due to the pro-apoptotic protein PAWR-mediated suppression of BCL2 [85]. Androgen-independent prostate cancer cell lines (PC-3 and DU 145) were tested where 3-AWFA treatment lead to conversion of cytosolic MAP1LC3B-I/LC3B-I to MAP1LC3B-I/LC3B-II (microtubule-associated protein-1 light chain 3β) and reduction of the autophagy substrate, SQSTM1 (sequestosome 1) [85]. The 3-AWFA molecule has also been reported separately by Rah et al. as a novel matrix metalloproteinase-2 (MMP-2) inhibitor. The authors investigated the mechanistic role of 3-AWFA as an extracellular Par-4 modulator on the invasion and angiogenesis of PC-3 and DU-145 cells compared to non-prostate cancer cells (HeLa and A549) [86]. Androgen receptor (AR) function suppression or blocking androgen signaling is an important therapy for androgen-dependent or independent therapy. Srinivasan et al. reported that treatment with WFA leads to apoptosis by a Par-4-dependent mechanism through caspase signaling and inhibition of NF-κB activity [57]. Nishikawa et al. reported that a treatment with 2 µM WFA resulted in cell death in androgen-independent prostate cancer cells (PC-3 and DU-145) compared to the androgen-sensitive cells (LNCaP) and to non-prostate normal fibroblasts (TIG-1 and KD). Compared to TIG-1 and LNCaP, the mRNA levels of c-Fos and 11 HSPs (heat-shock proteins) were increased in the WFA treated PC-3 and DU-145 cells but the expression of anti-apoptotic proteins c-FLIP (L) was found to be reduced [87].

### 5.4. Lung Cancer

Lung cancer is broadly classified into non-small cell lung cancer (NSCLC) representing 85% of all lung cancer cases, and small cell lung cancer (SCLC) (15%). Lung cancer is also classified based on the driver oncogenic mutation such as *EGFR* (epidermal growth factor receptor) mutation (20%), *ALK* (anaplastic lymphoma kinase) rearrangement (<5%), *KRAS* (20%) and *p53* mutations (50%) [88].

Hsu et al. have identified WFA as an effective NSCLC targeting agent via in silico screening. The authors have shown that WFA induces apoptotic cell death in both EGFR WT and mutant NSCLC cell lines with IC_50_ values ranging from 0.3–1.49 µM. Moreover, WFA inhibited the growth of H441 (EGFR WT and KRAS mutant c.35G > T) in vivo lung tumors in NOD/SCID (Non-obese Diabetic/severe combined immune deficiency; NOD.Cg-PrkdcscidIl2rgtm1Wjl/SzJl) mice [89]. Others have shown that WFA is effective for targeting KRAS mutant NSCLC cell lines in vitro e.g., A549 (c.34G > A), H3528 (c.34G > T) and H460 (c.183A > T) [90].

**Figure 4:**
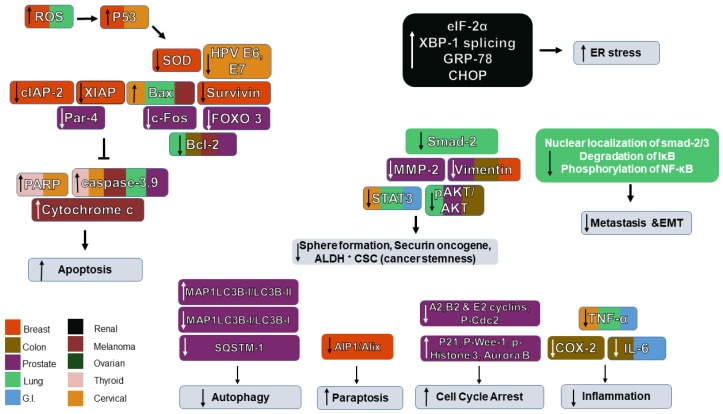
Schematic diagram of the role of Withaferin A in the signaling networks of various cancers and their associated protein regulation/dysregulation. “↑” denotes an increase, “↓” denotes decrease and “T” denotes inhibition.

In case of lung cancer treatment, WFA was reported to induce apoptosis in the NSCLC cell line A549 using annexin V/PI assay [91]. In addition, WFA inhibited the proliferation of A549 cells, as the number of cells in the G0/G1 phase was higher in treated cells. WFA caused a dose-dependent decrease in pAkt/Akt, the anti-apoptotic protein Bcl-2, and increases in Bax and cleaved caspase-3. Therefore, WFA was shown to have an anti-proliferative and pro-apoptotic action on A459 cells via suppression of PI3/Akt pathway [91] (Figure 4). Liu et al. have shown that WFA is selectively cytotoxic to A549 lung cancer cells at IC_50_ of 10 µM, whereas it is non-toxic to control normal lung cells WI-38 and PBMC. WFA increased cell apoptosis in annexin V/PI assay and Bax/Bcl ratio. Using JC-1 stain, WFA was found to induce a change in mitochondrial membrane potential indicating mitochondrial damage. WFA treated cells showed higher levels of caspase 3 and caspase 9 indicating the activation of mitochondrial pathway of apoptosis. WFA treatment showed a time-dependent increase in ROS production beginning 6 h after treatment and increasing until it reached a 5-fold increase after 24 h. Adding the anti-oxidant N-acetyl cysteine (NAC) to WFA treated A549 cells abrogated ROS production, apoptosis, and enhanced cell viability compared to groups treated with WFA alone, confirming that ROS production is an essential mechanism in WFA-mediated cytotoxicity [92].

Kyakulaga et al. have shown that WFA inhibits invasion and metastasis of NSCLC by inhibiting epithelial to mesenchymal transition (EMT). WFA was found to be cytotoxic in NSCLC cell lines with the metastatic cell line H1299 being more sensitive than the non-metastatic cell line A549. When the cells were treated with a sub-toxic dose of WFA, cell adhesion was reduced to 60–70% of the untreated cells. Moreover, WFA significantly reduced wound healing, migration and invasion of H1299 and A549 cells. WFA treatment abolished the *in vitro* induction of EMT using TGF-β (transforming growth factor β) and TNF-α. WFA treated cells showed no increase in EMT markers including: vimentin, claudin, fibronectin and snail. In addition, there was no change in cell morphology, loss of E-cadherin or increased vimentin expression. Mechanistically, WFA reduced the levels of smad-3 phosphorylation and nuclear localization of smad-2/3 which are known to mediate TGF-β induced EMT. In addition, WFA inhibited the degradation of Iκ-Bα phosphorylation and nuclear translocation of NF-κb leading to the inhibition of TNF-α-mediated EMT [93].

Kunimasa et al. showed that WFA in combination with glucose metabolism targeted therapy could be used as an effective treatment for tyrosine kinase inhibitor (TKI) drug-tolerant cancer cells. EGFR mutant lung cancer cell lines treated with gefitinib developed drug tolerance persisters (DTPs) characterized by increased senescence (CD133 ^low^) and stemness (marked by CD133 ^high^ population). Senescent cells show a phenotype called SASP (senescence-associated secretory phenotype) and can communicate with other cells through secreted factors. Conditioned media from gefitinib treated SASP increased the number of CD133^high^ cancer stem cells (CSCs). Gefitinib-tolerant DTPs were resistant to conventional cancer therapies such as cisplatin and pemetrexed. The group proposed using glucose metabolism targeting therapy (as senescent CD133 low cells are characterized by increased glucose metabolism) in combination with WFA for targeting CSCs. Glucose metabolism targeting therapies (e.g., phloretin) and WFA were found to possess an anti-tumor activity against gefitinib DTPs. Further, an in vivo gefitinib-induced DTP model was generated by treating a xenograft tumor with gefitinib until relapse occurred and the tumor continued to grow in the presence of the drug. Then, WFA and glucose transport inhibitor (phloretin) treatment was introduced causing a dramatic reduction in tumor size, suggesting that the combination of WFA and metabolism targeting therapies could be an effective therapeutic strategy against EGFR resistant lung cancer [94].

### 5.5. Ovarian Cancer

Amongst other cancers, WFA has also shown tremendous efficacy in ovarian cancer treatment particularly in combination therapy. Combination of WFA and cisplatin proved to be an effective treatment of refractory ovarian cancer (OC) by reducing the number of Aldehyde Dehydrogenase ALDH^+^ CSCs. Immunostaining showed that ALDH expression in the ovarian cortex was higher than its expression in ovarian surface epithelium (OSE) in borderline (BL) and high-grade (HG) ovarian tumors indicating the role of ALDH1 in invasion and metastasis of OC. In sphere formation assay, ALDH^+^ CSCs were isolated from the OC cell line A2780 spheroid formation was measured and found to be significantly reduced by WFA treatment at a dose of 1.5 µM. Cisplatin (CIS) treatment reduced the spheroid formation, albeit non-significantly. Cisplatin and WFA combination significantly reduced spheroid formation when compared to control, cisplatin only or WFA only treatment. In an orthotopic OC mouse model, WFA treatment significantly reduced ALDH^+^ CSC population, whereas Cisplatin treatment increased CSC population. The combination of CIS and WFA showed the highest reduction in ALDH^+^ CSCs (as shown by immunostaining and western blotting for ALDH1). WFA also reduced the levels of securin, an oncogene that is associated with cancer stemness. Cisplatin increased the levels of securin, indicating the enrichment of CSC population and this effect was reversed when the combination of CIS and WFA was used [19]. Kakar et al. have shown the WFA and DOXIL combination can be used for the elimination of ALDH^+^ CSCs responsible for relapse of OC patients [95].

### 5.6. Other Cancers

Apart from the major cancers described above, WFA has been reported to show potent anti-cancer properties in several other cancer types, such as gastric cancer, papillary and anaplastic thyroid cancers, cervical cancers, melanomas, renal carcinoma and promyelocytic leukemia.

In gastric cancer, WFA inhibited proliferation of human gastric adenocarcinoma (AGS) by inducing G2/M cell cycle arrest and apoptosis [96]. In addition to killing normal cancer cells, WFA was shown to target cancer stem cells and metastatic cancer cells. It was reported that in lymph node metastatic gastrointestinal cell line (UP-LN1), WFA reduced the CD44^high^/CD24^low^ floating (F) cell proliferation with greater apoptosis via downregulation of CXCR4/CXCL12 and STAT3/interleukin-6. The targeting ability of WFA on CSCs and mCSCs has been validated in NOD/SCID mouse xeno-transplantation [97].

In papillary and anaplastic thyroid cancers, combination of the multikinase-targeted inhibitor Sorafenib with WFA has been found to act synergistically via multiple mechanisms. PARP (Poly (ADP-ribose) polymerase cleavage, caspase-3 cleavage, BRAF/Raf-1 downregulation and inhibition of heat shock protein resulted from the combination therapy in vitro (B-CPAP, SW1736, human papillary and anaplastic thyroid cancer cell lines) [98].

In cervical cancer treatment, Munagala et al. showed for the first time that WFA restores the inactivation of the tumor suppressor p53 protein thus downregulating human papilloma virus (HPV) expressing E6/E7 oncogenes both in vitro and in vivo was found to be 0.45 ± 0.05 µM with altered expression levels of Bcl2, Bax, caspase-3, cleaved PARP. In addition, WFA lowered the levels of STAT3 and its phosphorylation at ^705^Tyr and ^727^Ser [99].

In case of melanoma, Mayola et al. tested WFA in four human melanoma cell lines and found WFA induced apoptotic cell death with IC_50_ ranging from 1.8 to 6.1 µM with the involvement of mitochondrial pathway. WFAs downregulated Bcl-2, with Bax mitochondrial translocation, cytochrome c release into the cytosol, activation of caspase 3 and 9 and fragmentation of DNA [100].

Endoplasmic reticulum stress was found to be the driving force in human renal carcinoma cells when treated with WFA. Dose-dependent WFA-induced apoptotic cell death in renal carcinoma kidney cell lines and induction of ER (endoplasmic reticulum) stress markers such as phosphorylation of eIF-2α (eukaryotic initiation factor-2α), XBP1 (X-box binding protein 1) splicing, upregulation of glucose-regulated protein (GRP)-78 and CHOP (CAAT/enhancer-binding protein-homologous protein). Mechanistically it was demonstrated by the pre-treatment of NAC (N-acetyl cysteine), the inhibition of WFA-mediated ER stress protein by ROS generated cell death [101].

Recently, Yu and co-authors investigated the action and mechanism of WFA on cancer cells with and without telomerase. Maintenance of telomere length by activation of telomerase or ALT (Alternative mechanism of Lengthening of Telomeres) led to overcoming replicative mortality by cancer cells and WFA was found to have stronger cytotoxic effects on ALT cells by telomere dysfunction, DNA damage, upregulation and inhibition of ALT-associated promyelocytic leukemia nuclear bodies in these cells. It was also found from computational and experimental analyses for effect on ALT mechanism, WFA led to Myc-Mad-mediated transcriptional suppression of an MRN complex protein (NBS-1) [102]. The overall effects of *WS* on different cancers are shown in Figure 4.

## 6. *WS* and Cancer Chemotherapy–Induced Toxicities.

The traditional chemotherapies induce many adverse effects including those affecting functions of several organs such as heart, liver, kidney, etc. In myocardial ischemia reperfusion (MI/R) injury WFA was found to increase cellular survival in simulated injury and in H_2_O_2_-induced cell apoptosis along with inhibition of oxidative stress. Thus, via upregulation of SOD2, SOD3, Prdx-1 by H_2_O_2_, WFA treatment leads to inhibition of the antioxidants and Akt-dependent improvement of cardiomyocyte caspase-3 [103]. Also, pre-treatment with WFA (10 mg/kg) induced a protective role as manifested by the lowering CYP450-mediated reactive metabolites resulting in oxidative stress in Bromobenzene-mediated liver and kidney damage. Oxidative stress and cytokines were reduced in addition to the prevention of mitochondrial dysfunction and restoring the balance between Bax/Bcl-2 in the WFA pre-treatment mice group [104]. Further, WFA reduced acetaminophen-induced liver toxicity in mice in Nrf2-dependent manner, which is a stress-responsive transcription factor and a validated chemoprevention target. In this study, Nrf2 signaling was induced by WFA in a non-canonical Keap-independent, Pten/PI3k/Akt-dependent manner [105]. Moreover, WFA decreased Cerulein-induced acute pancreatitis caused by oxidative stress and inflammation [106]. Lastly, WFA was found to induce antifibrotic activity in scleroderma by suppressing pro-inflammatory fibrosis involving TGF-β/Smad signaling and conversion to myofibroblasts, a FOXO3a-Akt-dependent NF-κβ/IKK-mediated inflammatory cascade [107]. A recent study involved the application of tumor targeting silver nanoparticles (Ag NP), which induce NP-related toxicity in macrophages. However, when NPs were administered along with the root extract of *WS* (35 mg/kg), the latter induced a significant reduction in toxic effects in rats [108].

Despite these basic and mechanistic studies, the potential of *WS* extracts as diet supplement has not been studied in the clinical realm except the single report that involved an open label prospective non-randomized trial on 100 breast cancer patients receiving chemotherapies that showed the potential of *WS* (used as complementary)-mediated decrease of treatment related fatigue and improved quality of life [109].

## 7. Concluding Remarks

The 2014–2023 World Health Organization (WHO) strategy aims to alleviate healthcare issues by providing traditional medicines as part of their affordable and effective alternative medicines to culturally diverse populations [110]. For a global implementation of these alternative herbal medicines, detailed and thorough evidence-based approaches should be executed to study their safety, efficacy and quality [111]. As reviewed herein, the recent clinical trials using randomized double-blind placebo control designs using *WS* extracts have shown that at specified dosage ranging from 200 mg/kg to 1000 mg/kg *WS* was not only effective, but most importantly at these dosages *WS* was safe and well tolerated. Further, numerous studies have shown anti-cancer efficacy using either *WS* or its major component WFA in human cancer cells lines and in murine models. The safety of *WS* in humans and the potential therapeutic efficacy seen in pre-clinical studies with the underlying diverse molecular pathways suggests the potential of *WS* and WFA use in patients with different cancers. There exist at least two different ways *WS* can be utilized against neoplastic diseases. First, given the safety record of *WS*, it can be used as an adjunct therapy that can aid in reducing the adverse effects associated with radio and chemotherapy due to its anti-inflammatory properties. Second, *WS* can also be combined with other conventional therapies such as chemotherapies to synergize and potentiate the effects due to radiotherapy and chemotherapy due to its ability to aid in radio- and chemosensitization, respectively. Taken together, all evidence to date indicate the potential of *WS* or WFA in cancer management. However, this needs to be validated in clinical studies prior to translation into the clinical realm.

## Figures and Tables

**Table 1:** Randomized Double-Blind Placebo Control Trials Involving *Withania Somnifera (WS*) in Interventional Studies

Disease, *n* of Subjects, Dose/Duration	Efficacy	Safety
Schizophrenia, *n* = 66, 1000 mg bid, 12 weeks	Medium effect sizes of favoring *WS* extracts (*WS*E) over placebo	Adverse events were mild and transient [9]
STAR (Strength Training Adaptations and Recovery) Trial, *n* = 19 (S500 vs. placebo), 500 mg/d, 12 weeks	Improved upper and lower-body strength, and supported a favorable distribution of body mass	Well tolerated clinically [10]
Idiopathic male infertility *n* = 50, 5 g/d, 90 days	Improves sperms parameters in idiopathic male infertility	Without causing adverse effects [11]
Subclinical Hypothyroid, *n* = 25, 600 mg daily	Compared to placebo, *WS* normalized serum TSH, serum triiodothyronine (T3) and thyroxine (T4) levels significantly in a subclinical double-blinded, randomized placebo-controlled trial.	Well tolerated [12]
Mild cognitive impairment (MCI), *n* = 50, 300 mg bid, 8 weeks	Improvement in overall cognitive function of adult patients with MCI	Effective in *WS* pre-treatment vs. post treatment [13]
Body weight management under chronic stress, *n* = 52, 300 mg bid, 6 weeks	Significant improvements in both primary and secondary measures	Well tolerated [14]

**Table 2:** Role of *WS* extracts of the different parts of the plant in cancers and the plausible pathways.

Extractant	Disease	Pathways or as Adjuvants
Whole Plant		
Methanol (75%)	Increase bone marrow cellularity; stem cell proliferation	Increase in total WBC (white blood cell) count; adjuvant during radiation therapy [30]
*WS* Root Extract		
Methanol (withanolide sulfoxide)	Gastric (AGS), breast (MCF-7), colon (HCT-116)	Suppressed TNF (tumor necrosis factor)-induced NF-kappa B activation [31]
Methanol	Neuroblastoma cells	alters basal and morphine-induced opioid receptor gene expression [32]
Methanol	Colon cancer	Chemotherapy through ‘Priming’ increases reactive oxygen species (ROS) [33]
Alcohol	Murine B16F1 melanoma	apoptosis through suppression of intrinsic pathway for carcinogenesis [34]
Ethanol	Spontaneous Estrogen Receptor-negative Mammary Cancer in MMTV/Neu Mice	significant decrease in CCL2 levels in mammary tumors [35]
Alcoholic extract	V79 Chinese hamster cells	Radiosensitizer [18]
DMSO	Human T leukemia cells	Immunogenic cell death; pro-apoptotic mechanism involves Ca^2+^ accumulation and generation of ROS [36]
Aqueous	Mouse Ehrlich ascites carcinoma	7.5 Gy gamma radiation combination synergistically [37]
WFA	HUVEC cells	Inhibition of NF-kappa B by interference with the ubiquitin-mediated proteasome pathway by increasing levels of poly-ubiquitinated proteins [38]
Water	Human malignant melanoma cells	Apoptotic body and nuclear blebbing [39]
Water	human MDA-MB-231 breast cancer cells	ROS-dependent mitochondria-mediated apoptosis [40]
Water	leukemic THP-1 cells and peripheral blood mononuclear cells (PBMCs)	Modulation of cancer cachexia associated inflammatory cytokines and cell death [41]
Leaves		
Methanol	Inflammatory disorders/cancer	Inhibition of NF-kappa B by preventing TNF-induced activation of Ikappa B kinase beta via thiol alkylation-sensitive redox mechanism [42]
Methanol	Breast, colon and liver cancer cell lines	Cell cycle arrest at S phase, increase in caspase 3 activity with induction of cell apoptosis [43]
Alcoholic	Glioma and YKG1 cell lines	induce senescence-like growth arrest and differentiation in glioma cells [44]
Water	Hepatocellular carcinoma	cell cycle arrest at G0/G1 and G2/M phases [45]
Water	Neuroinflammation	Microglial inactivation and migration via cell cycle arrest at G0/G1 and G2/M phase [46]
Water	Sarcoma, breast cell lines	activation of tumor suppressor proteins p53 and pRB, decrease in cyclin B1 and increase in cyclin D1, downregulation of MMP-3 and -9 [47]
Water	Glioma	Suppression of Tumor Growth of Intracranial Allograft of Glioma Cells by GFAP reduction and upregulation of mortalin and NCAM expression [48]
Alcohol	glioblastoma and neuroblastoma cells	oxidative stress and induction of differentiation [49]
Stems		
Methanol, ethanol and aqueous	Breast cancer (MDA-MB-231) and Vero cell lines	Cytotoxicity of the extracts were found and WFA was found to be the active component in both extracts [50]
Ethanol	HeLa, A549, BT474, MDA-MB-231, MDA-MB-453, T47D, MDA-MB-435S, G361, and WM 266.4 cells	Targeting Multidrug Resistance [51]
Fruit		
Methanol	Neurological disorders	BBB (blood-brain-barrier) permeability [52]
Methanol	HepG2	changes in the chromatin structure (fragmentation, uniform condensation) [53]

**Table 3:** Withaferin A (WFA), its role in cancer with the mechanism of actions.

Cancer Type	Mechanism of Action
Prostate cancer	Par-4-Dependent Apoptosis [57]
Myeloid leukemia HL-60 cells	Early ROS generation and mitochondrial dysfunction [58]
Breast cancer	FOXO3a (Forkhead box O3)- and Bim-dependent apoptosis [59]
Leukemic cells of lymphoid and myeloid origin	Mitochondrial apoptosis by activating p38 MAPK cascade [60]
Pancreatic cancer cells	Induction of proteasome inhibition and promotion the accumulation of ubiquitinated proteins, resulting in ER stress-mediated apoptosis [61]
Gliobastoma multiforme (GBM)	Orthotopic mouse model showed GBM neurosphere collapsed at nM concentrations [62]
Colorectal cancer cells	ROS-dependent mitochondrial dysfunction-mediated apoptosis [63]
